# In Silico Bioinformatics Analysis on the Role of Long Non-Coding RNAs as Drivers and Gatekeepers of Androgen-Independent Prostate Cancer Using LNCaP and PC-3 Cells

**DOI:** 10.3390/cimb45090459

**Published:** 2023-09-01

**Authors:** Mandisa Mbeje, Jeyalakshmi Kandhavelu, Clement Penny, Mmamoletla Kgoebane-Maseko, Zodwa Dlamini, Rahaba Marima

**Affiliations:** 1 SAMRC Precision Oncology Research Unit (PORU), DSI/NRF SARChI Chair in Precision Oncology and Cancer Prevention (POCP), Pan African Cancer Research Institute (PACRI), University of Pretoria, Hatfield 0028, South Africa; mandisa.mbeje@up.ac.za (M.M.); mw.kgoebane-maseko@up.ac.za (M.K.-M.); 2 Department of Medical Oncology, Faculty of Health Sciences, Steve Biko Academic Hospital, University of Pretoria, Hatfield 0028, South Africa; 3 Lombardi Comprehensive Cancer Center, Department of Oncology, Georgetown University Medical Center, Washington, DC 20057, USA; biojeya@gmail.com; 4 Department of Internal Medicine, Faculty of Health Sciences, School of Clinical Medicine, University of the Witwatersrand, Parktown 2193, South Africa; clement.penny@wits.ac.za; 5 Department of Anatomical Pathology, Faculty of Health Sciences, University of Pretoria, Hatfield 0028, South Africa

**Keywords:** prostate cancer, lncRNA, TERC, bioinformatics, miRNA, biomarker, ceRNA, TGF-β signaling

## Abstract

Prostate cancer (PCa) is the leading cancer in men globally. The association between PCa and long non-coding RNAs (lncRNAs) has been reported. Aberrantly expressed lncRNAs have been documented in each of the cancer “hallmarks”. Androgen signaling plays an important role in PCa progression. This study aimed to profile the aberrantly expressed lncRNAs in androgen-dependent (LNCaP) PCa compared to androgen-independent (PC-3) PCa cells. This was achieved by using a 384-well plate of PCa lncRNA gene panel. Differential expression of ±2 up or downregulation was determined using the CFX Maestro software v2.1. LncSEA and DIANA-miRPath were used to identify the enriched pathways. Telomerase RNA component (TERC) lncRNA was illustrated to participate in various tumourigenic classes by in silico bioinformatics analysis and was thus selected for validation using RT-qPCR. Further bioinformatics analysis revealed the involvement of differentially expressed lncRNAs in oncogenic pathways. Some lncRNAs undergo hypermethylation, others are encapsulated by exosomes, while others interact with several microRNAs (miRNAs), favouring tumourigenic pathways. Notably, TERC lncRNA was shown to interact with tumour-suppressor miRNAs hsa-miR-4429 and hsa-miR-320b. This interaction in turn activates TGF-β-signaling and ECM-receptor interaction pathways, favouring the progression of PCa. Understanding lncRNAs as competitive endogenous RNA molecules and their interactions with miRNAs may aid in the identification of novel prognostic PCa biomarkers and therapeutic targets.

## 1. Introduction

LncRNAs are known regulators of androgen-receptor (AR) signaling [1]. These RNA transcripts can bind to the enhancer region of the AR to transactivate it. Androgens are necessary for the development of the prostate gland and its normal physiological function. In PCa, androgens and AR signaling play a pivotal role in tumourigenesis [2]. An example of an AR-related lncRNA is CTBP1 antisense, which has regulatory functions in the transcriptional activity of AR. Furthermore, AR ubiquitination and protein degradation have also been shown to be enhanced by the lncRNA HOTAIR [3], indicating that the lncRNAs that regulate AR should be the primary focus of AR-related PCa studies.

LncRNAs are also involved in normal physiological functions such as cell differentiation, development, and the cell cycle [4]. They regulate gene expression at various levels and their mechanisms include transcriptional co-activation, post-transcriptional modification, chromatin remodelling, and protein inhibition [5]. Aberrantly expressed lncRNAs subsequently dysregulate gene expression regulation, which results in various diseases, including cancers. LncRNAs are cell-type and tissue-specific in their expression [6,7] and they competitively bind miRNAs for gene regulation in a complex competing endogenous RNA (ceRNA) network. 

This study aimed to profile differentially expressed lncRNAs and decipher their mechanisms between androgen-dependent (LNCaP) and androgen-independent (PC-3) cells. LNCaP cells express ARs and prostate-specific antigen (PSA). Moreover, their growth can be inhibited by androgen withdrawal, similar to human prostate adenocarcinoma. On the other hand, PC-3 cells have the highest metastatic potential [8]. These cells (PC-3) do not express ARs or PSA [9]. The AR signaling pathway is necessary for PCa cell growth and when PCa cells become androgen-independent, they no longer rely on androgens for growth. The molecular and cellular comparison between LNCaP cells and PC-3 cells is well described in the literature [9,10,11], and this study follows the same trend. While lncRNAs have been reported in the regulation of the AR signaling pathway, precise mechanisms remain largely to be elucidated. In this study, the lncRNAs’ expression between LNCaP and PC-3 cells was achieved by using a 384-well PrimePCR PCa-specific lncRNA gene array. The differentially expressed lncRNAs were further studied using in silico bioinformatics tools such as Long non-coding RNA-related Sets and Enrichment Analysis (LncSEA) and DIANA-miRPath. RT-qPCR was then used to validate the PrimePCR Array results. Through a comprehensive in silico analysis, this study demonstrated the role of lncRNAs in prostate cancer progression. 

## 2. Materials and Methods

### 2.1. Cell Culture

PC-3 cells (ATTC Catalogue number, CRL-1435) and LNCaP (ATCC Catalogue number, CRL-1740) were obtained from the American Type Culture Collection (ATCC). The PC-3 and LNCaP cells were grown in Dulbecco’s modified Eagle’s medium (DMEM):F12, supplemented with 10% fetal bovine serum (FBS) and 1% penicillin/streptomycin. The cells were kept in a humid incubator at 37 °C with 5% CO_2_ until they reached 70–80% confluency.

### 2.2. RNA Extraction and PrimePCR Array Analysis

RNA was extracted from the cultured LNCaP and PC-3 cells using the RNeasy Mini Kit (Qiagen, Frederick, MD, USA) (Catalogue number: 74104) following the manufacturer’s instructions. Genomic DNA (gDNA) was digested using an RNase-free DNase set (Qiagen, Frederick, MD, USA). Total RNA was eluted with 30 µL of RNase-free H_2_O and the RNA yield was measured using the NanoDrop ND-1000 spectrophotometer (NanoDrop Technologies, Wilmington, DE, USA). For the synthesis of cDNA, the ProtoScript^®^ II First Strand cDNA Synthesis Kit was used (New England Biolabs, Ipswich, MA, USA) (Catalogue number: E6560S). A Bio-Rad PCa-specific 384-well plate lncRNA PCR Array (Hercules, CA, USA) (Catalogue number: 12004237) was used to obtain the qPCR data (Table S1). This plate, with pre-dispensed primers lyophilized into the wells was loaded with the cDNA (LNCaP and PC-3) samples and POWERUP SYBR Master Mix (Thermo Fisher, Waltham, MA, USA) (Catalogue number: A25742). Due to Bio-Rad’s trademark control, the plate was run once. Each of the lncRNA genes was run in duplicate for LNCaP and PC-3 samples, respectively. An ABI QuantStudio™ 5 RT-qPCR system (Waltham, MA, USA) was used to run the qPCR reactions under the following conditions: 2 min at 95 °C, 15 s at 95 °C, 1 min at 95 °C, 15 s at 95 °C, 1 min at 60 °C, and 15 s at 95 °C. The array-generated data were quality-checked by analysing the Cq values of the assay quality controls. These included the positive PCR control, the reverse transcription control, the gDNA contamination control, and the RNA quality control. The array included three housekeeping genes, B2M, TBP, and HMBS. These genes were used for the calculation of ΔCq for each of the genes of interest, in CFX Maestro, where ΔCq was calculated as the Cq value of the gene of interest less than the Cq value of the reference gene. The difference between the ΔCq of the PC-3 and that of the LNCaP is represented by ΔΔCq value, where the fold change can be calculated by 2^(−ΔΔCq)^ [12]. This value is a representation of the level of expression of the lncRNA in the high tumour grade androgen-independent (PC-3) sample versus that in the low tumour grade androgen-dependent (LNCaP) sample. The ±2 upregulation or downregulation was used as a basis of target selection for downstream in silico bioinformatics analysis and RT-qPCR validation.

### 2.3. LncSEA Database v1.0

Annotation and enrichment analyses were performed using LncSEA v1.0 [13]. LncSEA database (http://bio.liclab.net/LncSEA/Analysis.php, (accessed on 16 November 2022) is a functional analysis tool that supports over 40,000 lncRNA reference sets and covers more than 50,000 lncRNAs [13]. LncSEA performs enrichment and annotation analyses of lncRNAs and outputs information such as lncRNA-interacting miRNAs. For this analysis, the differentially expressed lncRNAs from the CFX Maestro analysis were used as the input data in LncSEA, the parameters were set to include an adjusted *p*-value of 0.05 and a hypergeometric test *p*-value of 0.01. 

### 2.4. DIANA-miRPath v3.0 

The lncRNAs-miRNA relationship is an important aspect of gene regulation because lncRNAs can cause reduced regulatory effects of miRNAs on their mRNA targets [14]. The miRNA pathways were explored further using DIANA-miRPath v3.0 (http://www.microrna.gr/miRPathv3, accessednon 15 January 2023) [15]. This tool incorporates more than 600,000 experimentally supported miRNAs from DIANA-TarBase v7.0 [16] and allows for the visualization of the Gene Ontology (GO) and Kyoto Encyclopedia of Genes and Genomes (KEGG) pathways of the miRNA targets within the website [15].

#### 2.4.1. Kyoto Encyclopedia of Genes and Genomes (KEGG) Pathways

KEGG pathway enrichment analysis allows the mapping of biological pathways and molecular interactions associated with the genes of interest. The top 30 miRNAs with the lowest *p*-values (≤1.05 × 10^−6^) were selected and the default parameters of a microT threshold of 0.8 and a *p*-value threshold of 0.05 were applied.

#### 2.4.2. Gene Ontology 

GO is used to perform enrichment analysis to map biological processes, molecular functions, and cellular locations that are affected by the genes of interest. Similar to the KEGG analysis, 30 miRNAs with ≤1.05 × 10^−6^ *p*-values were selected, applying the default parameters. This was done to generate three heatmaps on the GO categories; namely, molecular function, biological process, and cellular component.

### 2.5. Cytoscape v3.9.1 

TERC lncRNA was observed to participate in various tumour-promoting processes as revealed by LncSEA and was selected for ceRNA network analysis. TERC miRNA gene targets were predicted using TargetScan v8.0 (http://www.targetscan.org/, accessed on 4 February 2023) [17] and TarBase v8.0 (https://dianalab.e-ce.uth.gr/html/diana/web/index.php?r=tarbasev8, accessed on 4 February 2023) [16]. Only the gene targets that were common among the databases and had a stringent TarBase prediction score of >0.9 were selected for the ceRNA network. The network was constructed and visualized using Cytoscape v3.9.1 [18].

### 2.6. Real-Time-Quantitative PCR (RT-qPCR)

RT-qPCR was performed for the validation of the PrimePCR data. Total RNA from the LNCaP and PC-3 cells was extracted using the RNeasy Mini Kit with DNase digestion (Qiagen, Hilden, Germany) following the instructions from the manufacturer. RNA samples were used to reverse transcribe cDNA using the ProtoScript^®^ II First Strand cDNA Synthesis Kit (New England Biolabs, Ipswich, MA, USA) using anchored d(T)_23_VN primers in an ABI 7500 Real-Time qPCR Instrument (ABI, Waltham, MA, USA). The gene target was TERC (accession number NR_001566.1), which was shown to be upregulated in the PrimePCR analysis and hypermethylated in LncSEA. Primer3 software v4.1.0 (https://primer3.ut.ee/, accessed on 17 November 2022) [19] was used to design the forward and reverse primers for TERC. These primers were synthesized by Integrated DNA Technologies (IDT) (Whitehead Scientific, Cambridge, MA, USA). GAPDH (NM_002046) was used as the housekeeping gene and the primer sequences were used: TERC; forward, TGCTCTAGAATGAACGGTGGA and reverse CCTAACTGAGAAGGGCGTAGG; GAPDH; forward, TGCACCACCAACTGCTTAGC and reverse GGCATGGACTGTGGTCATGAG. The following thermal cycling conditions were used: 10 min at 95 °C for denaturation, 40 cycles at 95 °C for 15 s, 1 min at 60 °C for data collection, 15 s at 95 °C, and 1 min at 60 °C for the dissociation stage. Fold changes were calculated using the 2^(−ΔΔCq)^ formula [12]. 

### 2.7. Statistical Analysis

The RT-qPCR data were collected for at least three independent experiments in triplicate and were analysed in GraphPad Prism v9.5.0 and expressed as means ± standard error of the mean (SEM). A one-sample t-test was performed to determine statistical significance, and a *p*-value < 0.05 was considered statistically significant.

## 3. Results

### 3.1. PrimePCR Array Analysis Using CFX Maestro

**Figure 1:**
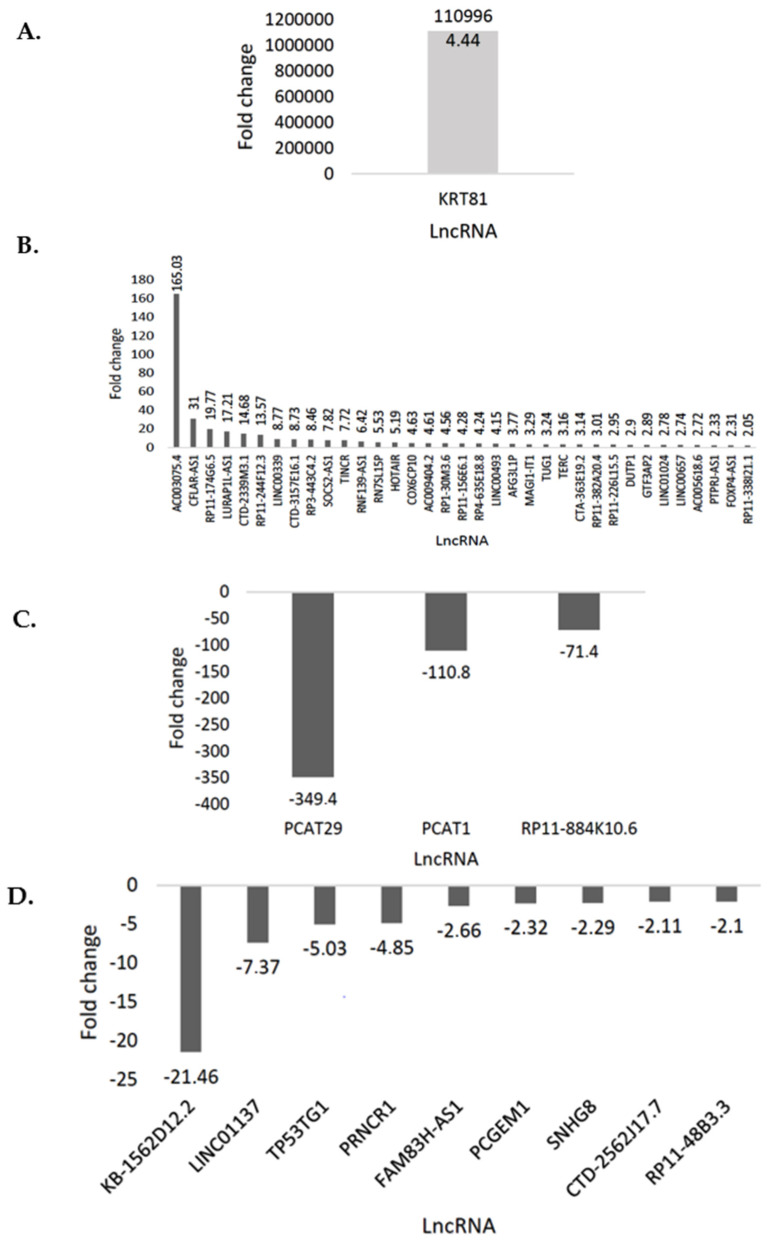
The differentially expressed lncRNAs in LNCaP and PC-3cell lines. (**A**) KRT81, the most upregulated lncRNA in PC-3 cells. (**B**) The top 35 upregulated lncRNAs in PC-3 cells. The transcripts presented in the histograms had an upregulation of at least 2-fold in the test PC-3 cells versus the control LNCaP cells. (**C**) The 3 most downregulated lncRNAs in PC-3 cells. (**D**) The 9 remaining downregulated lncRNAs in PC-3 cells with downregulation of at least negative 2-fold in the test PC-3 cells versus the control LNCaP cells.

The PrimePCR Array includes various control assays to ensure the reliability and reproducibility of the results. The array passed all the quality checks, including the positive PCR control, RT control, genomic DNA (gDNA) control assay, and RNA quality control assay. Of the 86 lncRNAs included in the lncRNA panel, 36 of these were upregulated (Figure 1A,B), including TERC, while 12 lncRNAs were downregulated (Figure 1C,D). 

**Figure 2:**
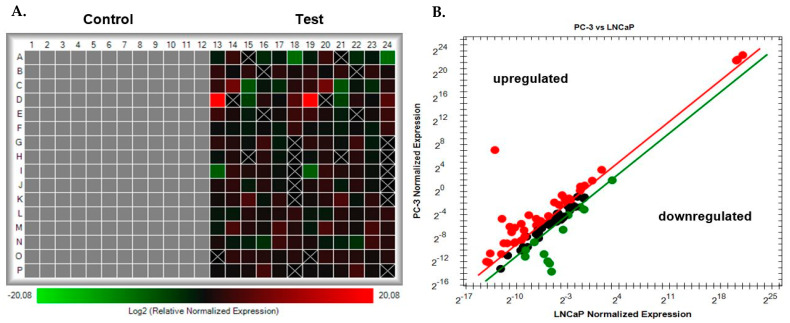
Expression of the differentially expressed lncRNAs in the PC-3 group vs. the LNCaP control group. (**A**) Heatmap showing the log2 relative normalized expression of lncRNAs. The colour intensity reflects the degree of upregulation (red) or downregulation (green), while ±20.08 represents the highest and the lowest value for up/downregulation. (**B**) Scatterplot that shows normalized expression between PC-3 and LNCaP lncRNAs. The regulation threshold was set to ±2. Upregulated lncRNAs are shown in red; downregulated genes are shown in green. The lncRNAs without significant gene expression changes are shown in black.

In this study, PCAT29, wells 18 and 24 (Figure 2A), is shown to be the most downregulated lncRNA with a fold change of −349.4. PCAT29 is a tumour suppressor that suppresses the metastasis of PCa cells [20]. The least downregulated lncRNA, RP11-48B3.3, had a regulation of −2.10. The most upregulated lncRNA was KRT81 with a fold change of 1,109,964.4. This lncRNA is part of the multigene keratin family that is expressed in multiple types of epithelia [21]. KRT81 is expressed in the hair cortex, and it is one of the main hair proteins. It is aberrantly expressed in this study, based on its function in PCa, and it has also been found to be upregulated in a breast cancer cell line and the lymph nodes of metastatic breast carcinoma [21]. The least upregulated lncRNA was RP11-338I21.1 with a regulation reading of 2.05. Amongst these upregulated lncRNAs are HOTAIR and TERC, which have been previously described to be upregulated in various cancers, including PCa [22,23]. HOTAIR is a well-documented lncRNA in the PCa literature [22]. On the other hand, there is a lack of studies regarding TERC lncRNA expression in PCa. Figure 2 shows a heatmap and a scatterplot of differentially expressed lncRNAs.

### 3.2. LncSEA Analysis of Differentially Expressed lncRNAs

**Figure 3:**
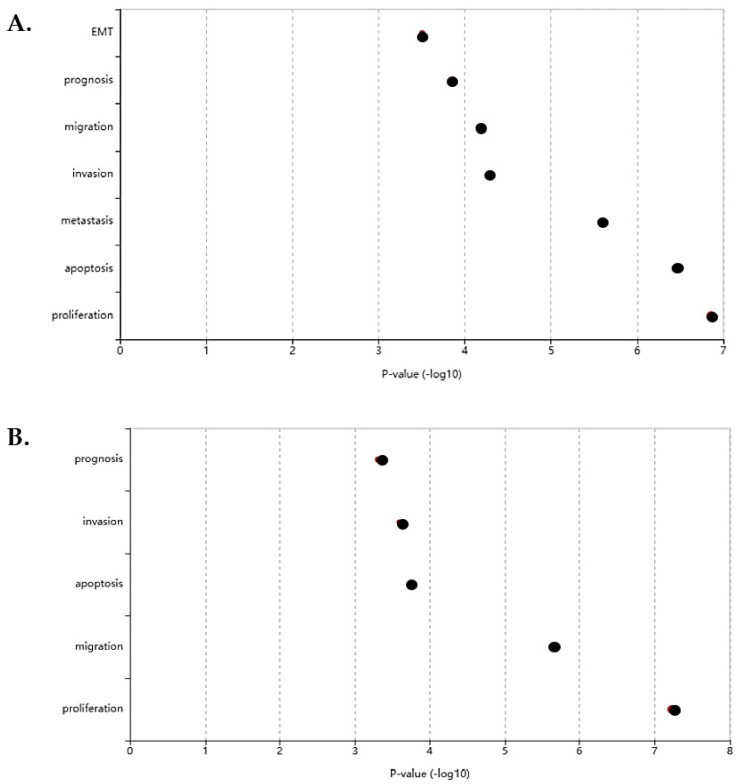
Graphs showing lncRNA-enriched cancer hallmarks and their associated *p*-value (−log10). (**A**) The upregulated lncRNAs were involved in seven cancer hallmarks. (**B**) The downregulated lncRNAs were associated with five cancer hallmarks, excluding metastasis and EMT.

The LncSEA database showed a total of 20 enriched upregulated lncRNAs and 9 enriched downregulated genes. Several lncRNAs were shown to be involved in the cancer hallmarks including epithelial-mesenchymal transition (EMT), invasion, and proliferation. In the cancer hallmark class of LncSEA, the proliferation hallmark shows the most enrichment with the highest −log10 *p*-value (Figure 3), indicating that differentially expressed lncRNAs are important for PCa progression. The EMT cancer hallmark showed the lowest −log10 *p*-value for the upregulated lncRNAs (Figure 3A), while the lowest −log10 *p*-value belonged to the prognosis cancer hallmark for the downregulated lncRNAs (Figure 3B).

**Figure 4:**
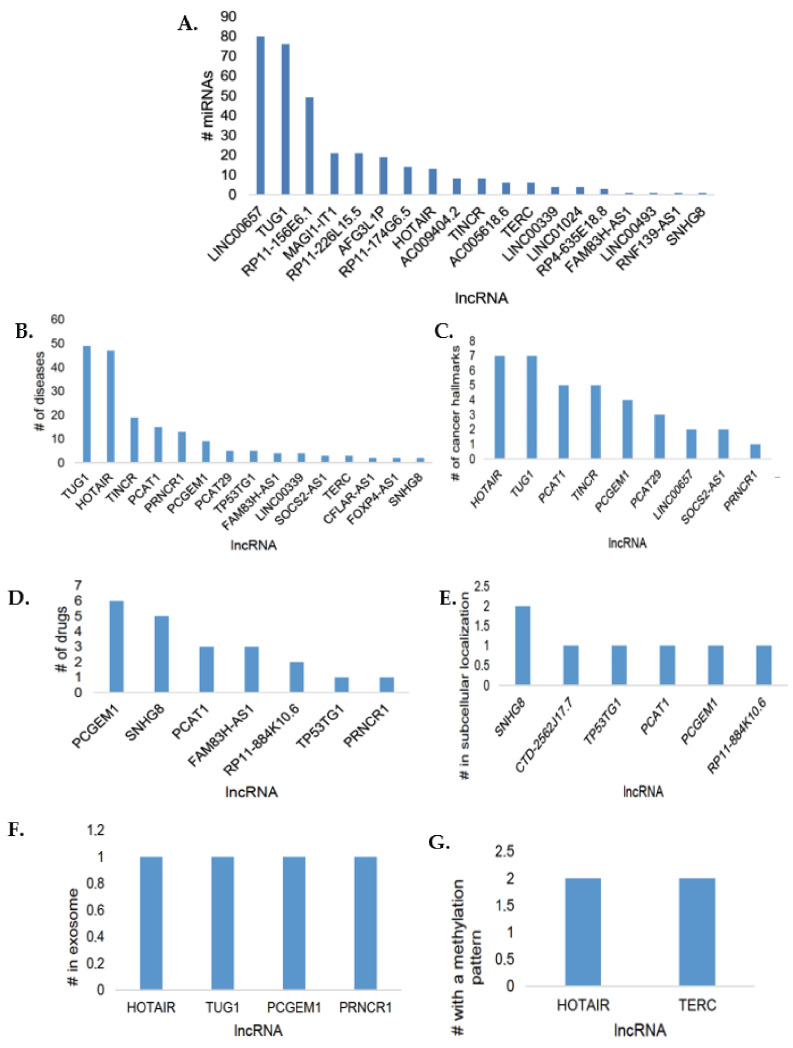
The differentially expressed lncRNAs in PC-3 cells in the LncSEA classes. (**A**) Twelve lncRNAs were shown to interact with miRNAs. (**B**) Fifteen lncRNAs were found to be associated with diseases including included PCa, glioma, and pre-eclampsia. (**C**) Nine lncRNAs were involved in cancer hallmarks. (**D**) Seven lncRNAs were shown to be drug targets in chemotherapy drugs such as Irinotecan. (**E**) Six lncRNAs were in the subcellular localization class of LncSEA. (**F**) Four lncRNAs were associated with the exosome, including TUG1. (**G**) Two lncRNAs were indicated to have a methylation pattern.

The miRNA class of LncSEA was demonstrated to be associated with the most lncRNAs, including HOTAIR, TUG1, LINC00657, and TERC (Figure 4A). This lncRNA/miRNA ceRNA network plays a pivotal role in PCa progression. Some of these miRNAs have tumour-suppressive properties and have been reported as potential PCa diagnostic biomarkers [24]. In LncSEA, TERC was shown to be hypermethylated (Figure 4G) and to interact with four members of the miR-320 tumour-suppressor family, namely hsa-miR-320a, hsa-miR-320b, hsa-miR-320c, and hsa-miR-320d. Two other tumour-suppressor miRNAs, hsa-miR-4429 and hsa-miR-338-3p, were also shown to interact with TERC. These findings suggest that the TERC lncRNA undergoes hypermethylation and in turn represses the tumour-suppressor miRNAs, thus promoting uncontrolled PCa progression to androgen independence. 

### 3.3. DIANA-miRPath Analysis of the Differentially Expressed lncRNAs and Their Associated miRNA Interactions

#### 3.3.1. KEGG Pathway Analysis

**Figure 5:**
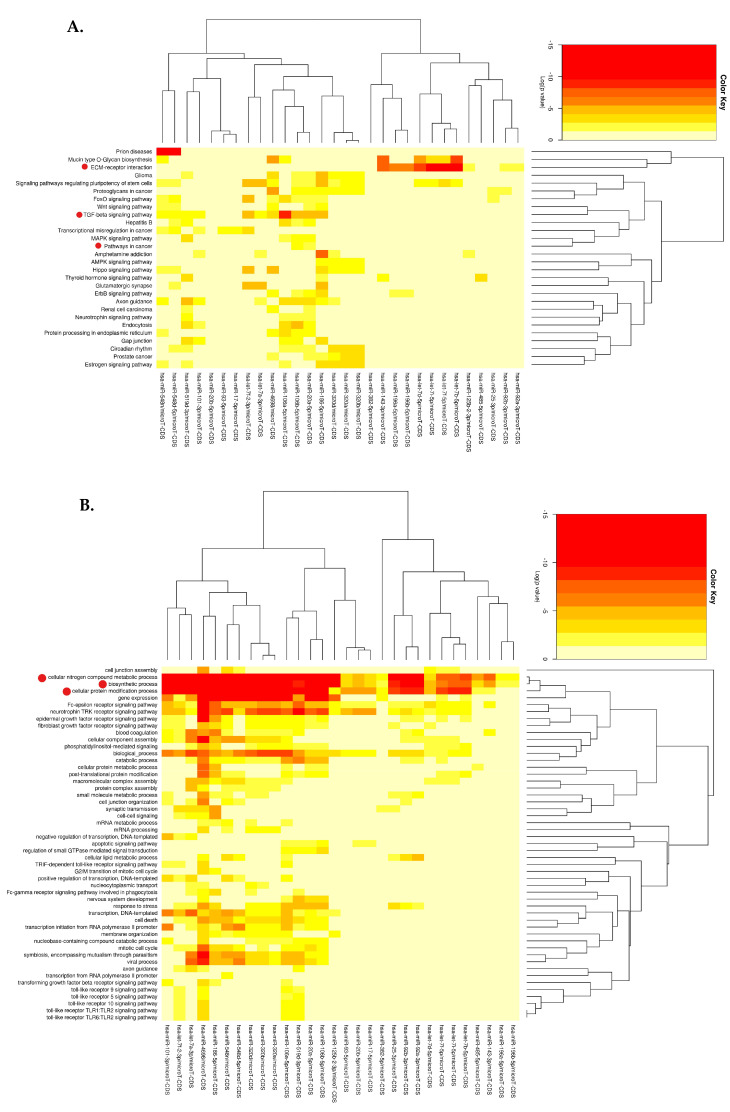

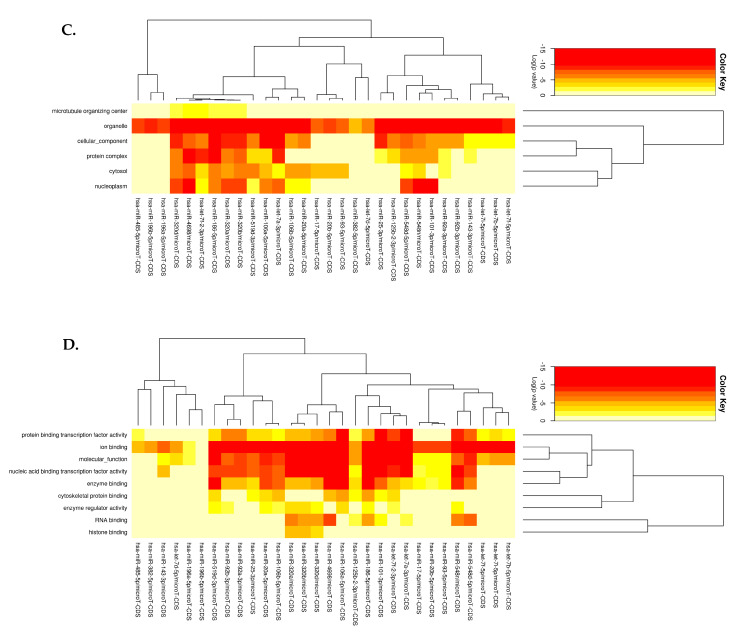
Heatmaps of the lncRNA-associated miRNAs and the pathways and biological processes they are involved in; *p*-value < 0.05; microT < 0.8; Fischer’s Test analysis method with fold discovery rate (FDR) correction. Lower log(*p*-values) indicate higher enrichment and a deeper red colour. (**A**) The KEGG pathways and their associated miRNAs include PCa, ECM-receptor binding, and the TGF-beta signaling pathways (denoted by the red dot). (**B**) The biological processes GO subcategory includes the biosynthetic, cellular nitrogen compound metabolism, and cellular protein modification processes and their associated miRNAs (denoted by the red dot). (**C**) The cellular component GO subcategory and the (**D**) molecular function GO subcategories and the miRNAs that are linked to them.

The lncRNA–miRNA relationship was further analysed using DIANA-miRPath v3.0 (Figure 5). The KEGG pathway analysis and biological process GO analysis showed 28 enriched pathways. The KEGG pathway analysis (Figure 5A) revealed that the extracellular matrix (ECM)–receptor interaction is most enriched by the hsa-miR-let7 family. Prion disease shows the most enrichment by hsa-miR-548d-5p and hsa-miR-458n. Prion disease is caused by the human prion protein. Interestingly, the upregulation of this protein has been identified to promote various cancers and aid their resistance to cancer therapies [25]. Hsa-miR-106a-5p shows higher enrichment of the TGF-β signaling pathway which is involved in tumourigenesis. PCa and other pathways related to PCa were also identified (Figure 5A).

#### 3.3.2. GO Enrichment Analysis

The most enriched GO categories are the biosynthetic process, cellular nitrogen compound metabolic process, and cellular protein modification process (Figure 5B). These processes are enriched by various miRNAs including the hsa-miR-106 a and b family and the hsa-miR-320 family, which are involved in various cancers, including PCa. Figure 5C,D show the cellular component and molecular function GO subcategories, respectively. 

### 3.4. Bioinformatics Analysis and lncRNA–miRNA-mRNA Competing Endogenous RNA (ceRNA) Network

**Figure 6:**
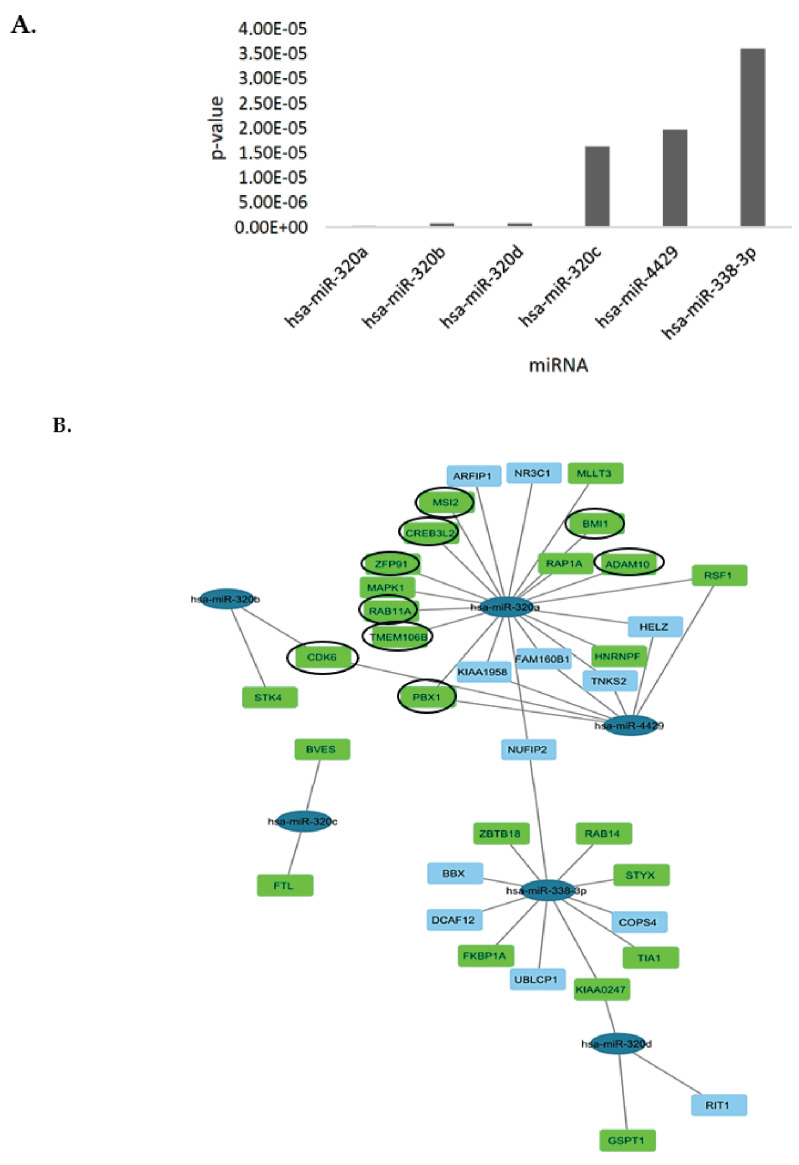
The ceRNA network using TERC-associated miRNAs. (**A**) CeRNA network basis of the miRNAs linked to TERC lncRNA based on the *p*-value. (**B**) The ceRNA network with the miRNAs is represented in dark blue ellipses, and the gene targets are represented in blue rectangles. The cancer progression targets are shown in green rectangles. The circled green targets are linked to PCa progression specifically.

The in silico analyses revealed that TERC hypermethylation was associated with several miRNAs including the hsa-miR-320 family (Figure 6A), which are important regulators of oncogenic pathways. A ceRNA network visualized the TERC-associated mRNA targets (Figure 6B) and revealed several gene targets that were predicted to interact with the TERC-associated miRNAs. Nine of these genes were involved in tumour progression in PCa including ADAM10, which enhances PCa metastasis (Figure 6B).

### 3.5. Validation of the Selected lncRNA Using Real-Time Quantitative PCR (RT-qPCR)

**Figure 7:**
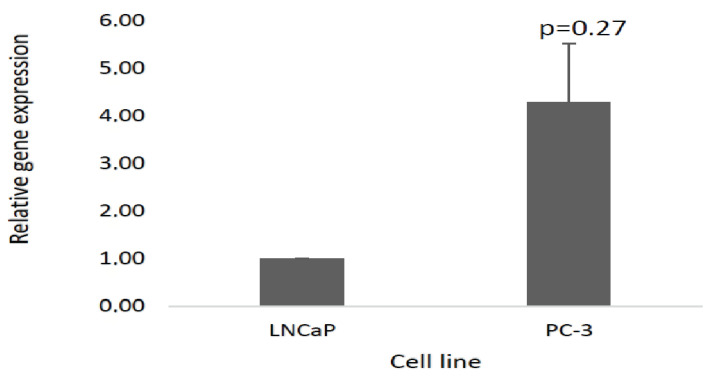
The validation of TERC lncRNA gene expression using RT-qPCR in the PC-3 test compared to LNCaP control cells. A 3.29 TERC upregulation is observed in PC-3 cells compared to LNCaP. The error bars denote the SEM. All the reactions were run in triplicate, three independent times. However, *p* > 0.05 was observed.

Based on the PrimePCR data and bioinformatics analysis, one of the upregulated lncRNAs, TERC (Figure 1B), was selected for downstream RT-qPCR. TERC is reportedly overexpressed in various tumours including PCa [26]. However, precise TERC oncogenic mechanisms remain poorly understood, especially in PCa. TERC expression was shown to be insignificantly upregulated in androgen-independent PC-3 cells (*p*-value > 0.05) (Figure 7). Though TERC is shown to be involved in various tumourigenic pathways by in silico bioinformatics analysis, its precise mechanisms in PCa progression and androgen signaling remain to be fully defined. 

## 4. Discussion

In this study, it was revealed that several lncRNAs were differentially expressed between androgen-dependent (LNCaP) and androgen-independent (PC-3) cells. Some of these lncRNAs were shown to be hypermethylated and some were encapsulated by extracellular vesicles, enriching several oncogenic pathways. Furthermore, it was revealed that these lncRNAs interact with miRNAs, favouring PCa progression. In a study by Zhang et al., KRT81 knockdown was shown to regulate interleukin-8 to inhibit the progression of metastatic melanoma [27]. KRT81 has also been shown to contribute to breast cancer cell migration and invasion [21]. In PCa, KRT81 is understudied, however, in this study, KRT81 was shown to be aberrantly expressed and associated with PCa metastasis (Figure 1A). 

The lncRNA HOTAIR is well described in the cancer biology literature [28,29,30]. HOTAIR is often implicated in PCa and has been shown to promote the progression of PCa into castration-resistant PCa (CRPC) [3]. HOTAIR is repressed by androgens, which results in its upregulation upon androgen deprivation therapies (ADTs). It prevents the ubiquitination process of ARs. This subsequently prevents protein degradation by inhibiting the binding of the androgen receptor to E3 ubiquitin ligase MDM2 [3]. Therefore, its upregulation in the highly metastatic PC-3 cells, as shown in this current study, is substantiated by previous studies. 

TUG1 and LINC00657 are frequently studied lncRNAs in various cancers, except PCa. TUG1 was upregulated with a fold change of 3.24 and LINC00657 with a fold change of 2.74 (Figure 1B). The lncRNA TUG1 has been shown to be upregulated in many malignancies [31] and its overexpression is closely linked to cancer prognosis [32]. TUG1 competitively binds and sponges the miRNA called miR-26a in PCa. The resulting effect is PCa cell proliferation, indicating that TUG1 may be an oncogenic lncRNA in PCa [33]. TUG1 is a cancer-associated fibroblast (CAF)-derived exosomal lncRNA [34]. Exosomal lncRNAs have increased stability as they are protected by the exosomal membrane structures. It is for this reason that TUG1 can be targeted as a prognostic and diagnostic biomarker [35]. The lncRNA LINC00657 is a linked with DNA damage [36] and it is carcinogenic in gastric cancer [37]. Interestingly, this lncRNA is tumour-suppressive in glioblastoma by miR-190a-3p sponging [38]. This implies that one lncRNA may be oncogenic or tumour-suppressive in different cancers, thus serving as a double-edged sword. This warrants the necessity for studying the precise mechanisms of lncRNAs in various tumour grades and in different cancers.

PCGEM1 upregulation has been shown to drive aggressive PCa [39]. PCGEM1 enhances AR-mediated gene activation, thus promoting PCa progression. In this study, PCGEM1 is shown to be downregulated (Figure 1D). A similar result was demonstrated by Srikantan et al., where PCGEM1 was shown to be expressed in LNCaP cells and undetectable in PC-3 cells [40]. This observation is further evidence of PCGEM1’s androgen dependence.

Hypermethylation can drive oncogenesis [41] and suppress the expression of genes. The LncSEA analysis revealed that TERC was hypermethylated (Figure 4G) and that it interacts with several miRNAs, especially tumour-suppressive miRNAs (Figure 4A). Although this study is the first to report TERC hypermethylation and sponging of the tumour-suppressor miRNA-320 family in PCa, similar TERC oncogenic mechanisms have been reported in gastric cancer, where TERC was shown to sponge miR-423-5p, thus promoting the proliferation of gastric cancer [42]. TERC upregulation in PC-3 cells is further supported by RNA-sequencing data, where in normal prostate epithelia, its expression is −16.61 [43].

LncRNA–miRNA–mRNA regulatory networks are critical for cancer development and progression [44]. This is facilitated by miRNA sponging, which is the main mechanism for lncRNA–miRNA interactions. This interaction involves miRNA response elements (MREs) and ceRNAs [45], which function by competing with miRNAs to inhibit gene expression, and their sponging effect is the basis of a network of various biochemical processes. LncRNAs regulate miRNAs and miRNAs can also regulate lncRNAs. The degradation of lncRNAs is mediated by miRNAs through DNA methylation as well [45]. Therefore, lncRNA–miRNA relationships are important for normal physiological function and development, and aberrantly expressed lncRNAs can negatively affect these miRNA interactions, leading to cancer. 

TERC is shown to interact with the hsa-miR-320 family, hsa-miR-338-3p, and hsa-miR-4429. The decreased expression of the hsa-miR-320 family has been shown in various tumours including PCa [24]. This indicates that TERC hypermethylation correlates with the downregulated expression of the hsa-miR-320 family in PCa. TERC lncRNA facilitates PCa cell metastasis, migration, proliferation, and migration by sponging the hsa-miR-320 family and inhibiting their tumour-suppressive abilities. This TERC/hsa-miR-320 regulatory axis may potentially lead to the understanding of PCa prognostic mechanisms and may be targeted by novel therapeutics. The miR-320 family is involved in PCa prognosis, indicating the diagnostic potential of this family. Moreover, serum levels of these miRNAs between patients who had a high tumour stage and patients who had a low tumour stage were significantly different [24]. The miR-320 family is further implicated in the inhibition of EMT, cell proliferation, and metastasis [46]. The miR-320 family is reportedly downregulated in the tumourigenesis process in numerous studies [47,48], further indicating that it functions as a tumour suppressor. Furthermore, hsa-miR-338-3p and hsa-miR-4429 also have tumour-suppressive properties in glioblastoma [49], cervical cancer [50], and also in PCa [51]. This implicates TERC as an oncogenic lncRNA that sponges tumour-suppressing miRNAs, promoting cancer progression.

In normal physiology, hsa-miR-338-3p was shown to target the *RAB23* gene, thus suppressing tumourigenesis. *RAB23* gene is a member of the Fold discovery rate RAS oncogene family [52], and it was not predicted to interact with hsa-miR-338-3p in this current study. This miRNA interacted with *RAB14* instead, which promotes the proliferation and invasion of lung cancer [53]. *RAB14*’s relevance in cancer proliferation is also substantiated by evidence that upon overexpression, the Wnt and AKT signaling pathways are activated in gastric and ovarian cancers [53].

In PCa, hsa-miR-4429 suppresses cell proliferation [51] and it is reported to sensitize cervical cells to irradiation [50], demonstrating its tumour-suppressive role. This is done by inactivating the Wnt/β-catenin pathway, which induces PCa cell invasiveness and maintains cancer cell stemness. Cyclin-dependent kinase 6 (CDK6) was shown to be a target for this miRNA in the current study. The literature supports that this miRNA is involved in suppressing colorectal cancer progression via the miR-500a-3p/CDK axis. This does not mean that CDK6 exclusively interacts with hsa-miR-500a-3p, as there may be several other miRNAs interacting with the same gene target, as shown in Figure 6B. 

The ceRNA network shows that some genes are targeted by more than one miRNA in the network. The nuclear fragile X mental retardation protein interacting protein 2 (NUFIP2), for example, interacts with both hsa-miR-320a and hsa-miR-338-3p. Several of these TERC-associated mRNA targets from the analysis are linked to the progression of PCa, such as a disintegrin and metalloproteinase domain-containing protein 10 (ADAM10), Musashi RNA binding protein 2 (MSI2), and cyclin-dependent kinase 6 (CDK6) amongst others. The analysis also showed that hsa-miR-320c is the only miRNA that does not have a common mRNA target with another miRNA in this ceRNA network, warranting further elucidation, particularly in PCa. 

**Figure 8:**
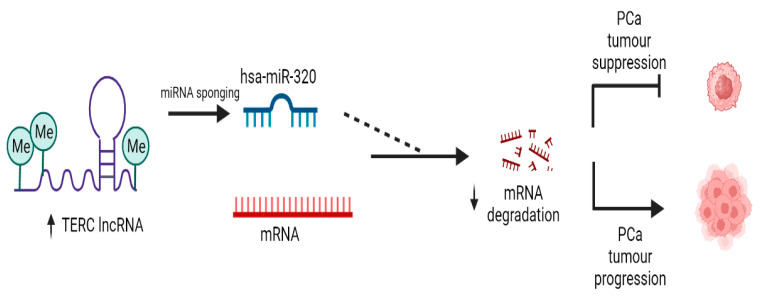
Schematic summarizing the mechanism of TERC facilitating PCa progression. The upregulation of TERC lncRNA competitively binds tumour-suppressive hsa-miR-320 to inhibit oncogenic mRNA degradation. Subsequently, the tumour is not repressed, and PCa cells grow and proliferate (created with BioRender.com).

Hypermethylated TERC interacts with tumour-suppressive miRNAs, thus promoting PCa progression. Along with the understanding that lncRNAs may function as ceRNAs sponging miRNAs, it can be concluded that TERC may be an oncogenic lncRNA, which sponges tumour-suppressive miRNAs to promote PCa progression (Figure 8). 

Limitations of this study have also been observed. Even though these arrays have proprietary/trademark control, they were run once, each of the 86 lncRNAs in duplicate. Additional prostate cell lines such as the normal cell line (HprEpC) and other PCa cell lines (MDA-PCa-androgen sensitive, DU-145-androgen resistant) representing different tumour grades and androgen signaling status could in future be included to further decipher TERC oncogenic mechanisms. Nonetheless, understanding complex lncRNAs’ regulatory mechanisms in cancer biology, treatment and drug discovery requires enhanced computational tools such as machine learning algorithms and molecular docking. Computational procedures are often designed to complement laboratory-based assays in studying complex systems such as llncRNA/protein interactions (LPIs) [54]. While treatment resistance is a major challenge in PCa, computational capabilities that enable in silico approaches are advancing in improving drug discovery. Molecular dynamics (MD) is an important tool in drug development and design. Unlike the traditional MD methods, more complex hybrid classical/quantum mechanical (QM) methods, MD simulations offer enhanced insights into ligand/receptor interactions [55]. MD approaches offer an array of opportunities to understand molecular and structural mechanisms. Thus, combining these computational procedures with other in silico approaches and validated experiments can improve the opportunities to identify more effective next-generation drugs to treat PCa. 

## 5. Conclusions and Future Perspectives

The differential expression of lncRNAs in various cancers including PCa has been documented. The purpose of this study was to compare lncRNA expression between androgen-sensitive and androgen-independent PCa cells. The comparison of these cell lines has been demonstrated previously in the literature [10,11]. Although there were limitations to this study, such as running the Prime gene array once with each lncRNA gene in duplicate, the findings of this study illustrate a significant role played by lncRNAs in PCa progression. In particular, to our knowledge, this is the first study to reveal TERC tumour-promoting mechanisms through hypermethylation in PCa. Additionally, lncRNA–miRNA–mRNA interactions revealed an interplay between several miRNAs and gene targets, demonstrating the complexity of gene regulation by lncRNAs. A significant research gap exists in fully understanding lncRNAs mechanisms in cancer progression. 

TERC lncRNA and other lncRNAs can be explored as prognostic biomarkers in PCa progression. Similarly, LINC00668 and long non-coding (lnc)-SAYSD1-1 lncRNAs may be used to discriminate ERG/not-ERG PCa subtypes. With high PCa tumour heterogeneity and various molecular subtypes, TERC can be used to better stratify PCa patients and predict prognoses [56]. Furthermore, it has been reported that extracellular vesicles (EVs) modulate the sensitivity of cancer cells to chemotherapeutic agents by delivering molecularly active lncRNAs such as TERC [57]. Such mechanisms involve the lack of oncogene translational repression by sponging of tumour-suppressor miRNAs by lncRNAs such as TERC. TERC may be a potential target in understanding and addressing PCa treatment resistance. Thus, studies associated with differential gene expression, associated molecules, and the pathways that are affected hold potential for a greater understanding of lncRNAs-medicated PCa progression. 

## Data Availability

The data presented in this study are available in the figures of this manuscript and the supplementary materials. Raw data can be made available upon request.

## Supplementary Materials

The following supporting information can be downloaded at: https://www.mdpi.com/article/10.3390/cimb45090459/s1, Table S1: Layout of the PrimePCR array plate.
