# Supplementary material for: In Silico Bioinformatics Analysis on the Role of Long Non-Coding RNAs as Drivers and Gatekeepers of Androgen-Independent Prostate Cancer Using LNCaP and PC-3 Cells
Source: Curr Issues Mol Biol. 2023 Sep 1;45(9):7257–74. doi: 10.3390/cimb45090459 (PMC10528188; doi:10.3390/cimb45090459)
Supplement: Supplementary file 1 [file cimb-45-00459-s001.zip › cimb-2565815-supplementary.pdf]

Table S1: Layout of the PrimePCR array plate

	1	2	3	4	5	6	7	8	9	10	11	12	13	14	15	16	17	18	19	20	21	22	23	24
A	PCGEM1	SOCS2-AS1	PCA3	PRNCR1	CBR3-AS1	PCAT29	PCGEM1	SOCS2-AS1	PCA3	PRNCR1	CBR3-AS1	PCAT29	PCGEM1	SOCS2-AS1	PCA3	PRNCR1	CBR3-AS1	PCAT29	PCGEM1	SOCS2-AS1	PCA3	PRNCR1	CBR3-AS1	PCAT29
B	RP1-30M3.6	GNG12-AS1	LINC00493	RP11-619J20.1	RP4-635E18.8	HMGN3-AS1	RP1-30M3.6	GNG12-AS1	LINC00493	RP11-619J20.1	RP4-635E18.8	HMGN3-AS1	RP1-30M3.6	GNG12-AS1	LINC00493	RP11-619J20.1	RP4-635E18.8	HMGN3-AS1	RP1-30M3.6	GNG12-AS1	LINC00493	RP11-619J20.1	RP4-635E18.8	HMGN3-AS1
C	RN7SL15P	AC003075.4	RP11-884K10.6	RP11-390P2.4	TP53TG1	RP11-382A20.4	RN7SL15P	AC003075.4	RP11-884K10.6	RP11-390P2.4	TP53TG1	RP11-382A20.4	RN7SL15P	AC003075.4	RP11-884K10.6	RP11-390P2.4	TP53TG1	RP11-382A20.4	RN7SL15P	AC003075.4	RP11-884K10.6	RP11-390P2.4	TP53TG1	RP11-382A20.4
D	KRT81	RP4-724E16.2	KB-1562D12.2	TERC	RP11-545E17.3	CFLAR-AS1	KRT81	RP4-724E16.2	KB-1562D12.2	TERC	RP11-545E17.3	CFLAR-AS1	KRT81	RP4-724E16.2	KB-1562D12.2	TERC	RP11-545E17.3	CFLAR-AS1	KRT81	RP4-724E16.2	KB-1562D12.2	TERC	RP11-545E17.3	CFLAR-AS1
E	RP3-443C4.2	FGD5-AS1	SNHG8	RP11-556O9.2	KCNQ1OT1	TINCR	RP3-443C4.2	FGD5-AS1	SNHG8	RP11-556O9.2	KCNQ1OT1	TINCR	RP3-443C4.2	FGD5-AS1	SNHG8	RP11-556O9.2	KCNQ1OT1	TINCR	RP3-443C4.2	FGD5-AS1	SNHG8	RP11-556O9.2	KCNQ1OT1	TINCR
F	SNHG19	RP11-379H18.1	RP11-759F5.1	lnc-FAM83G-3	XIST	RNF139-AS1	SNHG19	RP11-379H18.1	RP11-759F5.1	lnc-FAM83G-3	XIST	RNF139-AS1	SNHG19	RP11-379H18.1	RP11-759F5.1	lnc-FAM83G-3	XIST	RNF139-AS1	SNHG19	RP11-379H18.1	RP11-759F5.1	lnc-FAM83G-3	XIST	RNF139-AS1
G	AFG3L1P	LINC01024	RP11-46C24.7	LINC00339	RP11-48B3.3	B2M	AFG3L1P	LINC01024	RP11-46C24.7	LINC00339	RP11-48B3.3	B2M	AFG3L1P	LINC01024	RP11-46C24.7	LINC00339	RP11-48B3.3	B2M	AFG3L1P	LINC01024	RP11-46C24.7	LINC00339	RP11-48B3.3	B2M
H	AC006994.1	FOXP4-AS1	RP11-146E13.4	MAGI1-IT1	RP4-591B8.2	HMBS	AC006994.1	FOXP4-AS1	RP11-146E13.4	MAGI1-IT1	RP4-591B8.2	HMBS	AC006994.1	FOXP4-AS1	RP11-146E13.4	MAGI1-IT1	RP4-591B8.2	HMBS	AC006994.1	FOXP4-AS1	RP11-146E13.4	MAGI1-IT1	RP4-591B8.2	HMBS
I	PCAT1	HOTAIR	LINC00657	PTPRJ-AS1	SCHLAP1	TBP	PCAT1	HOTAIR	LINC00657	PTPRJ-AS1	SCHLAP1	TBP	PCAT1	HOTAIR	LINC00657	PTPRJ-AS1	SCHLAP1	TBP	PCAT1	HOTAIR	LINC00657	PTPRJ-AS1	SCHLAP1	TBP
J	RP3-523E19.2	COX6CP10	CTD-2562J17.7	CTA-363E19.2	WAC-AS1	PAQ1	RP3-523E19.2	COX6CP10	CTD-2562J17.7	CTA-363E19.2	WAC-AS1	PAQ1	RP3-523E19.2	COX6CP10	CTD-2562J17.7	CTA-363E19.2	WAC-AS1	PAQ1	RP3-523E19.2	COX6CP10	CTD-2562J17.7	CTA-363E19.2	WAC-AS1	PAQ1
K	RP11-121A14.2	RP11-226L15.5	RP11-174G6.5	RP11-473I1.5	RP11-156E6.1	PAQ2	RP11-121A14.2	RP11-226L15.5	RP11-174G6.5	RP11-473I1.5	RP11-156E6.1	PAQ2	RP11-121A14.2	RP11-226L15.5	RP11-174G6.5	RP11-473I1.5	RP11-156E6.1	PAQ2	RP11-121A14.2	RP11-226L15.5	RP11-174G6.5	RP11-473I1.5	RP11-156E6.1	PAQ2
L	RP11-196G18.22	BCDIN3D-AS1	GTF3AP2	RN7SL277P	RP11-338I21.1	gDNA	RP11-196G18.22	BCDIN3D-AS1	GTF3AP2	RN7SL277P	RP11-338I21.1	gDNA	RP11-196G18.22	BCDIN3D-AS1	GTF3AP2	RN7SL277P	RP11-338I21.1	gDNA	RP11-196G18.22	BCDIN3D-AS1	GTF3AP2	RN7SL277P	RP11-338I21.1	gDNA
M	RP11-119B16.2	LURAP1L-AS1	DUTP1	UBA6-AS1	CTD-2339M3.1	PCR	RP11-119B16.2	LURAP1L-AS1	DUTP1	UBA6-AS1	CTD-2339M3.1	PCR	RP11-119B16.2	LURAP1L-AS1	DUTP1	UBA6-AS1	CTD-2339M3.1	PCR	RP11-119B16.2	LURAP1L-AS1	DUTP1	UBA6-AS1	CTD-2339M3.1	PCR
N	RP11-67L2.2	RP11-363E7.4	FAM83H-AS1	LINC01137	CTD-3157E16.1	RQ1	RP11-67L2.2	RP11-363E7.4	FAM83H-AS1	LINC01137	CTD-3157E16.1	RQ1	RP11-67L2.2	RP11-363E7.4	FAM83H-AS1	LINC01137	CTD-3157E16.1	RQ1	RP11-67L2.2	RP11-363E7.4	FAM83H-AS1	LINC01137	CTD-3157E16.1	RQ1
O	NHS-AS1	AC009404.2	TUG1	AC005618.6	PSMD6-AS2	RQ2	NHS-AS1	AC009404.2	TUG1	AC005618.6	PSMD6-AS2	RQ2	NHS-AS1	AC009404.2	TUG1	AC005618.6	PSMD6-AS2	RQ2	NHS-AS1	AC009404.2	TUG1	AC005618.6	PSMD6-AS2	RQ2
P	CTD-2010I16.1	ZNF767P	RP11-98I9.4	RP11-244F12.3	NRSN2-AS1	RT	CTD-2010I16.1	ZNF767P	RP11-98I9.4	RP11-244F12.3	NRSN2-AS1	RT	CTD-2010I16.1	ZNF767P	RP11-98I9.4	RP11-244F12.3	NRSN2-AS1	RT	CTD-2010I16.1	ZNF767P	RP11-98I9.4	RP11-244F12.3	NRSN2-AS1	RT
